# Integration of neural architecture within a finite element framework for improved neuromusculoskeletal modeling

**DOI:** 10.1038/s41598-021-02298-9

**Published:** 2021-11-26

**Authors:** Victoria L. Volk, Landon D. Hamilton, Donald R. Hume, Kevin B. Shelburne, Clare K. Fitzpatrick

**Affiliations:** 1 Micron School of Materials Science and Engineering, Boise State University, Boise, ID USA; 2 Mechanical and Biomedical Engineering, Boise State University, 1910 University Drive, MS-2085, Boise, ID 83725-2085 USA; 3 Center for Orthopaedic Biomechanics, University of Denver, Denver, CO USA

**Keywords:** Biomedical engineering, Physiology, Systems biology, Computer modelling

## Abstract

Neuromusculoskeletal (NMS) models can aid in studying the impacts of the nervous and musculoskeletal systems on one another. These computational models facilitate studies investigating mechanisms and treatment of musculoskeletal and neurodegenerative conditions. In this study, we present a predictive NMS model that uses an embedded neural architecture within a finite element (FE) framework to simulate muscle activation. A previously developed neuromuscular model of a motor neuron was embedded into a simple FE musculoskeletal model. Input stimulation profiles from literature were simulated in the FE NMS model to verify effective integration of the software platforms. Motor unit recruitment and rate coding capabilities of the model were evaluated. The integrated model reproduced previously published output muscle forces with an average error of 0.0435 N. The integrated model effectively demonstrated motor unit recruitment and rate coding in the physiological range based upon motor unit discharge rates and muscle force output. The combined capability of a predictive NMS model within a FE framework can aid in improving our understanding of how the nervous and musculoskeletal systems work together. While this study focused on a simple FE application, the framework presented here easily accommodates increased complexity in the neuromuscular model, the FE simulation, or both.

## Introduction

Human movement requires complex interactions between the nervous system and musculoskeletal system. The nervous system generates electrical signals in the brain that are transmitted through the spinal cord to the neuromuscular junction. At the junction, the electrical signal is converted to a muscle activation that generates a muscle force causing motion at the joints. A major limitation in studying human systems, particularly the nervous system and the neuromuscular junction, is the challenge of performing in vivo experiments. In humans, studies investigating the neuromuscular junction are oftentimes difficult or infeasible to perform, particularly due to ethical concerns^1^. Recording electrical activity at the cellular level can be dangerous to perform in humans and although there are types of external recordings, such as electroencephalography (EEG) and electromyography (EMG), these recordings occur at the brain and muscle level and do not provide cellular level data about what is occurring at the neuromuscular junction. This is where computational models, specifically fully predictive neuromusculoskeletal (NMS) models, can play a significant role. NMS models include components of both the nervous and musculoskeletal systems necessary to fully study the neuromuscular junction and resulting movement in a manner that is not possible in vivo.

In the field of biomechanics, musculoskeletal simulations are used to perform analyses capable of assessing geometry, loading and boundary conditions, and material properties in situations that cannot be measured within a living organism^2^. Two key types of musculoskeletal models are rigid body and finite element (FE) models. Rigid body simulations are useful for simulating musculoskeletal dynamics and calculating joint kinematics from experimental data^3^. For more complex problems, such as detailed representation of the joints that include soft tissue geometries and material properties, FE analyses are often more useful. FE simulation environments (e.g. FEBio, febio.org; Abaqus, Simulia) can be used for both rigid-body simulations and more complex FE simulations. However, neither of these approaches involve neural control to drive the musculoskeletal models.

Neural data-driven models that use EMG as the input are an exception to this lack of neural control in driving musculoskeletal models^4–9^. They are beneficial for in-depth studies to quantify musculoskeletal function and control^8^ via neural drive, or common synaptic input, to the spinal cord and muscles^4^. However, these EMG driven models inform force production based only on decomposition of discharge times and no other neural anatomy. They also only operate in a feed-forward method that does not have the feedback from the musculoskeletal system to the nervous system required for the nervous system to adapt during movement.

Alternatively, fully predictive NMS models utilize a pool of motor neurons^10–12^ or neural networks with motor neurons, Renshaw cells, and interneurons^13–17^ to simulate a neural command that generates a simulated muscle force used in a musculoskeletal model. This means that the signal being converted into muscle force is based upon a variety of neural factors such as anatomy, types of ion channels, and connectivity between different neurons, which can all be modified to study their effects. Neural factors can be varied throughout the simulation that make the overall outputs representative of the adaptation that occurs in the body. This is a key benefit of fully predictive models, rather than studying musculoskeletal function from a specific neural drive^1^.

NEURON is an open-source, Python-based simulation environment that is used to create models ranging from individual neurons to networks of neurons^18^. Previously developed models in NEURON have been able to accurately simulate the neural drive to muscles^19^, but do so in a single motor unit that would not represent in vivo muscle contraction. Motor unit recruitment and rate coding are the two ways in which muscle forces in skeletal muscle are varied and controlled^20^. If a neuromuscular model does not exhibit these two functions, then it cannot replicate muscle force or movement generation in an in vivo manner. Recruitment is the concept that not all motor units (a motor neuron and all the muscle fibers it innervates) are active at a given time, but instead are recruited in an orderly manner^20^. Motor units are recruited in size order from smallest to largest, following Henneman’s size principle^21^, where ones that generate smaller forces are recruited first followed by larger force producing motor units. Rate coding involves a proportional relationship between stimulation intensity and discharge rate, such that as the intensity of a stimulus increases, so does the rate of discharging action potentials^20^. All motor neurons have a recruitment threshold, below which no action potential will be generated. For stimuli that are above the recruitment threshold there exists a linear relationship between the level of injected current and the resulting discharge rate. The discharge rate will continue to increase with increased current intensity until the peak rate is achieved. After this point, there is little variation in discharge rate, even with a continued increase in excitatory drive. NEURON by itself simulates the electrical impulses representative of movement, but does not simulate the actual movement. By integrating NEURON with a FE environment, we can create a comprehensive multiscale simulation framework with the ability to model movement from initial neural command generated in the brain at the cellular level through to the resulting muscle contraction necessary for joint movement at the human systems level.

In this study, we develop a fully predictive NMS model that uses an embedded neural architecture within a FE environment to simulate muscle activation and force. We demonstrate the ability of this integrated framework to implement motor unit recruitment and rate coding capabilities in the human physiological range. This is accomplished by integrating finite element (Abaqus, Simulia, Providence, RI) and NEURON simulation environments and is demonstrated here using a motor neuron pool innervating a soleus muscle in a simple musculoskeletal model. A combination of complex neuronal networks with musculoskeletal modeling is needed for multifaceted analyses and simulation of the interaction between the nervous and musculoskeletal systems. The novel framework developed in this study has been implemented here in a simple FE model. However, this framework can accommodate increased complexity in the neuromuscular model, the FE simulation, or both, facilitating the development of multi-system models that may be used in future work for investigation of neurodegenerative or neurodevelopmental conditions.

## Results

**Figure 1:**
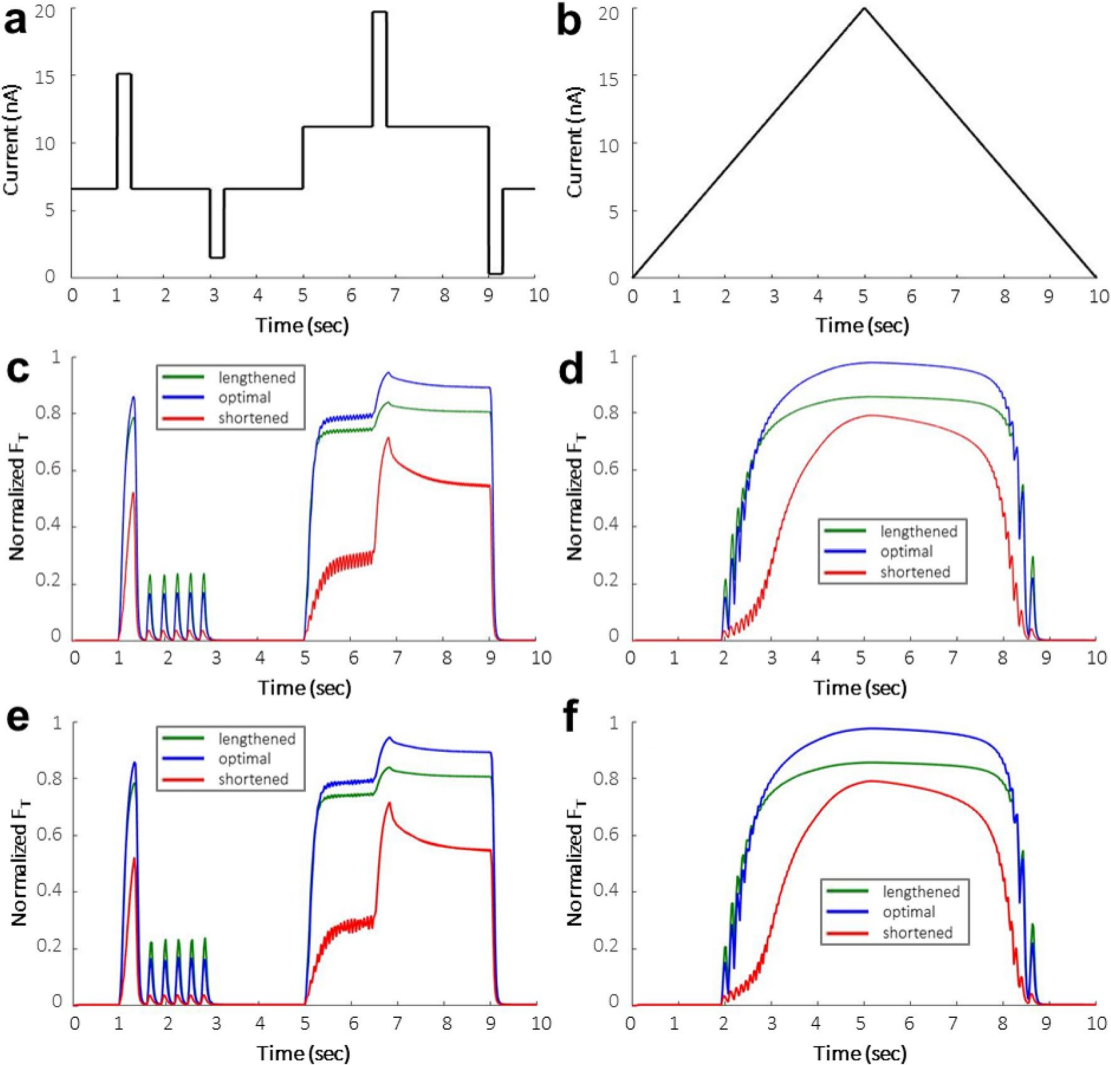
(**a**, **b**) Input activation profiles from Kim^19^ implemented in the single neuron NMS model to show software integration. (**c**, **d**) Neuromuscular muscle force results from Kim^19^ (Figs. 3B, 4B), reproduced here using publicly available data from ModelDB^32^. (**e**, **f**): Muscle force outputs from FE NMS simulations at lengthened, optimal, and shortened muscle lengths.

The muscle force outputs from the single motor neuron FE NMS simulation at three muscle lengths—0 mm, − 8 mm, and − 16 mm—or lengthened, optimal, and shortened muscle states, respectively, reproduced the results reported by Kim^19^ (Fig. 1). The root mean square error (RMSE) between the NEURON force predictions of the neural model by itself and the integrated FE NMS model at the optimal muscle length are 0.0513 N and 0.0492 N for the reproduction of Kim Figs. 3b and 4b^19^, respectively. The RMSE at the lengthened and shortened muscle states are 0.0467 N and 0.0407 N for Fig. 3b and 0.0424 N and 0.0307 N for Fig. 4b, respectively^19^. These RMSE values verify the effective integration of the NEURON and FE software environments.

The total time for a 10.0 s simulation in the FE NMS model framework was approximately 12 min for a single motor neuron. Of that, 8 min was the time taken for the NEURON component of the simulation and 4 min for the Abaqus FE component.

### Verification of in vivo neural behavior

**Figure 2:**
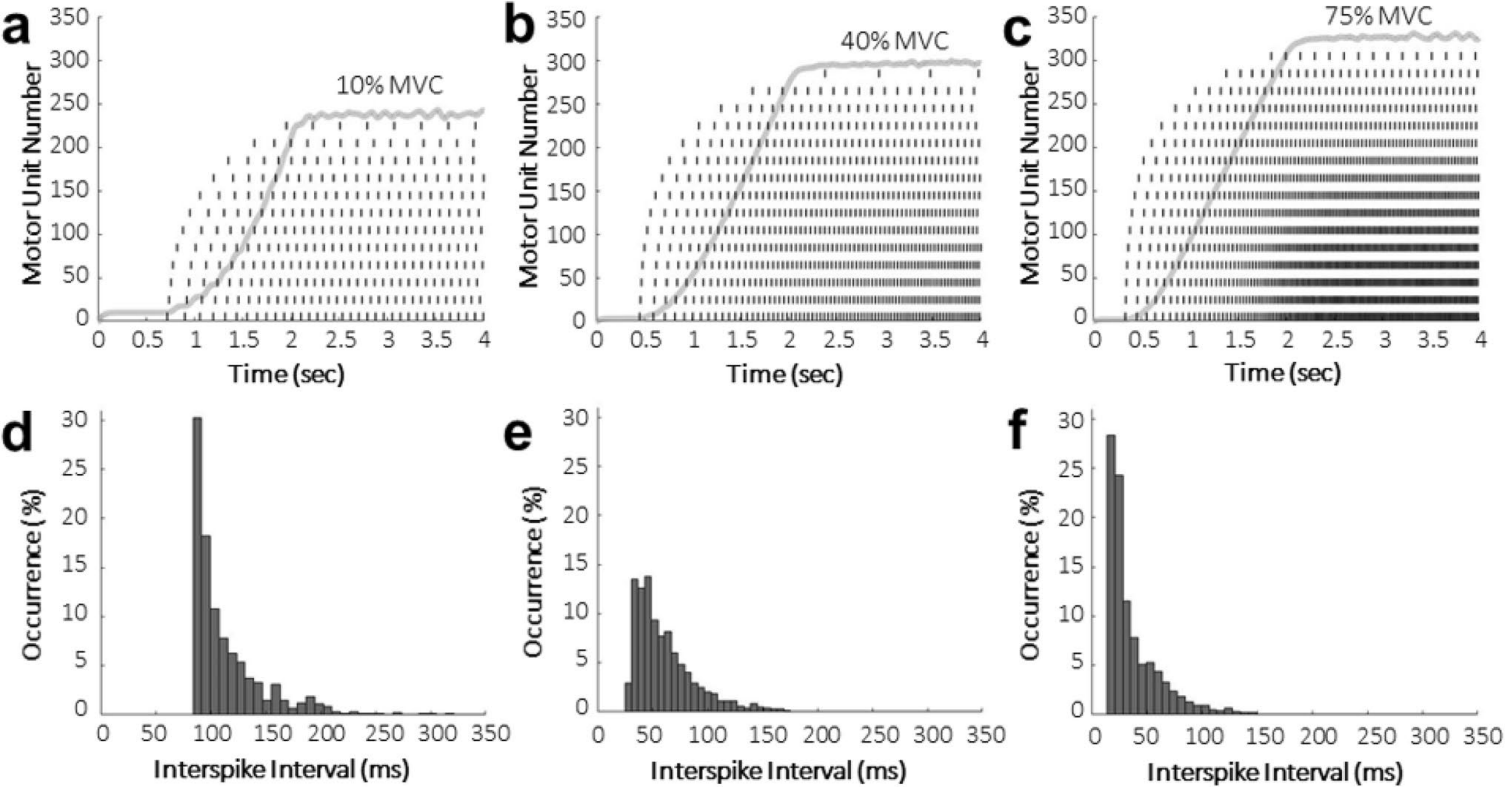
(**a**–**c**) Discharge times for every 20th motor unit (dashes) with resulting output muscle force (solid line). Motor unit 1 is the smallest and motor unit 310 is the largest, with an exponential size distribution. The stimulation profile increased linearly for two seconds until reaching the peak amplitude corresponding to that %MVC, after which point it was held constant for two seconds. (**d**–**f**) Interspike interval measurements between each subsequent discharge for every motor unit through the length of the simulation. Intervals with less than five occurrences were not included in the figure for visualization.

**Figure 3:**
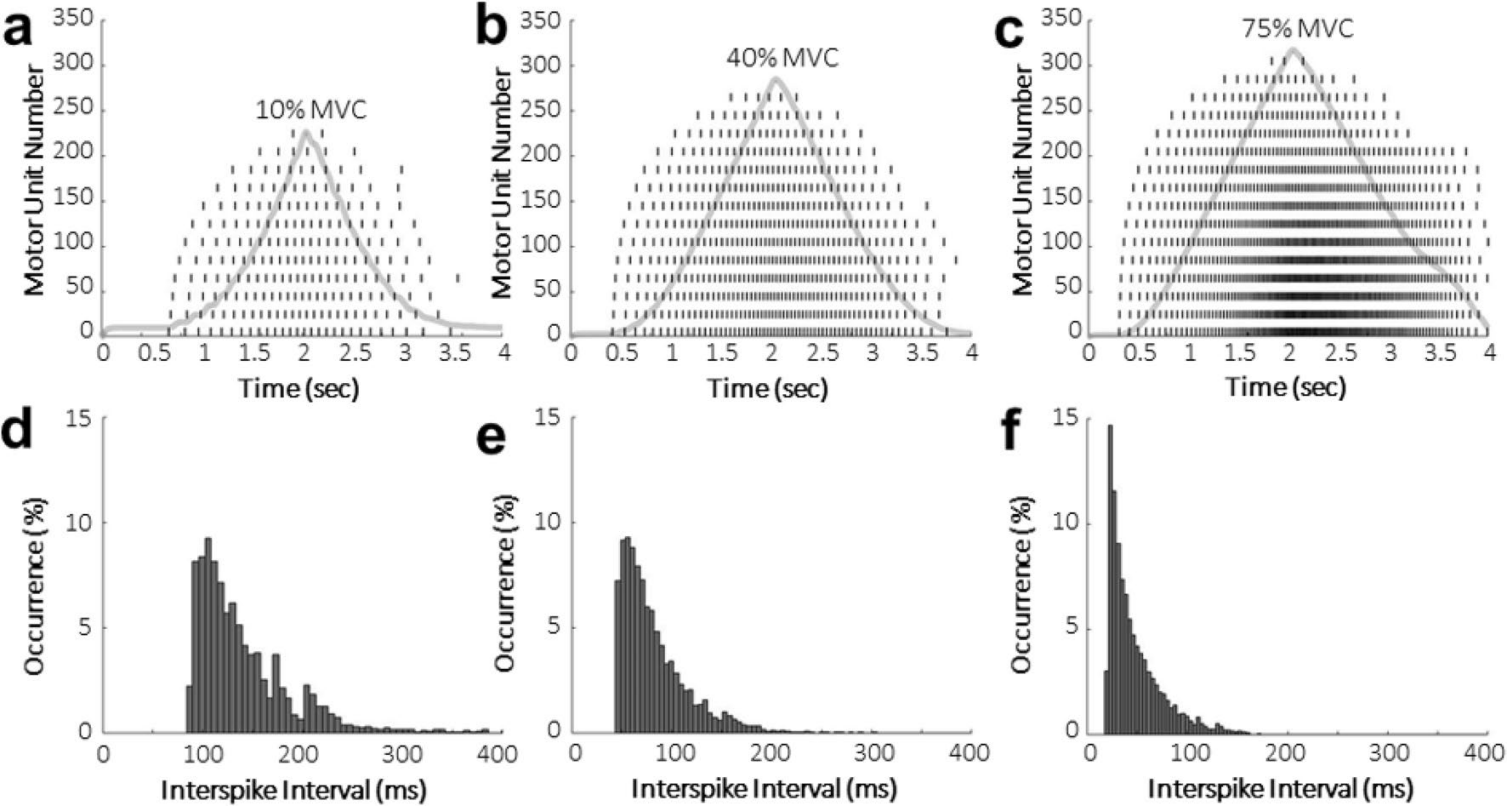
(**a**–**c**) Discharge times for every 20th motor unit (dashes) with resulting output muscle force (solid line). The stimulation profile increased linearly for two seconds until reaching the peak amplitude corresponding to that %MVC, after which point it decreased linearly back to baseline over two seconds. (**d**–**f**) Interspike interval measurements between each subsequent discharge for every motor unit through the length of the simulation. Intervals with less than five occurrences were not included in the figure for visualization.

The integrated FE NMS model scaled to a neuronal network of 310 motor units effectively demonstrated motor unit recruitment for two stimulation profiles at three muscle force levels (Figs. 2, 3). Motor unit recruitment follows an exponential distribution where smaller motor units are recruited before larger motor units. The resulting muscle forces increased linearly until the last motor unit of that simulation was recruited, which is representative of physiologically accurate muscle behavior at greater force levels^20^. The interspike interval plots (Figs. 2d–f, 3d–f) show a decrease in time between successive action potential discharges, or increased discharge rate, with an increase in stimulation intensity and correspond to an increase in percent maximum voluntary contraction (%MVC).

All motor units had recruitment thresholds between > 0 and 75% MVC and followed an exponential distribution. The average (± standard deviation) motor neuron diameter in the neuronal network of 310 motor units was 61.58 ± 13.08 μm. The average (± standard deviation) motor neuron diameter for motor units recruited between 0 – 30% MVC was 57.77 ± 8.25 μm. The average (± standard deviation) motor neuron diameter for motor units recruited between 50 and 75% MVC was 94.60 ± 3.10 μm.

**Figure 4:**
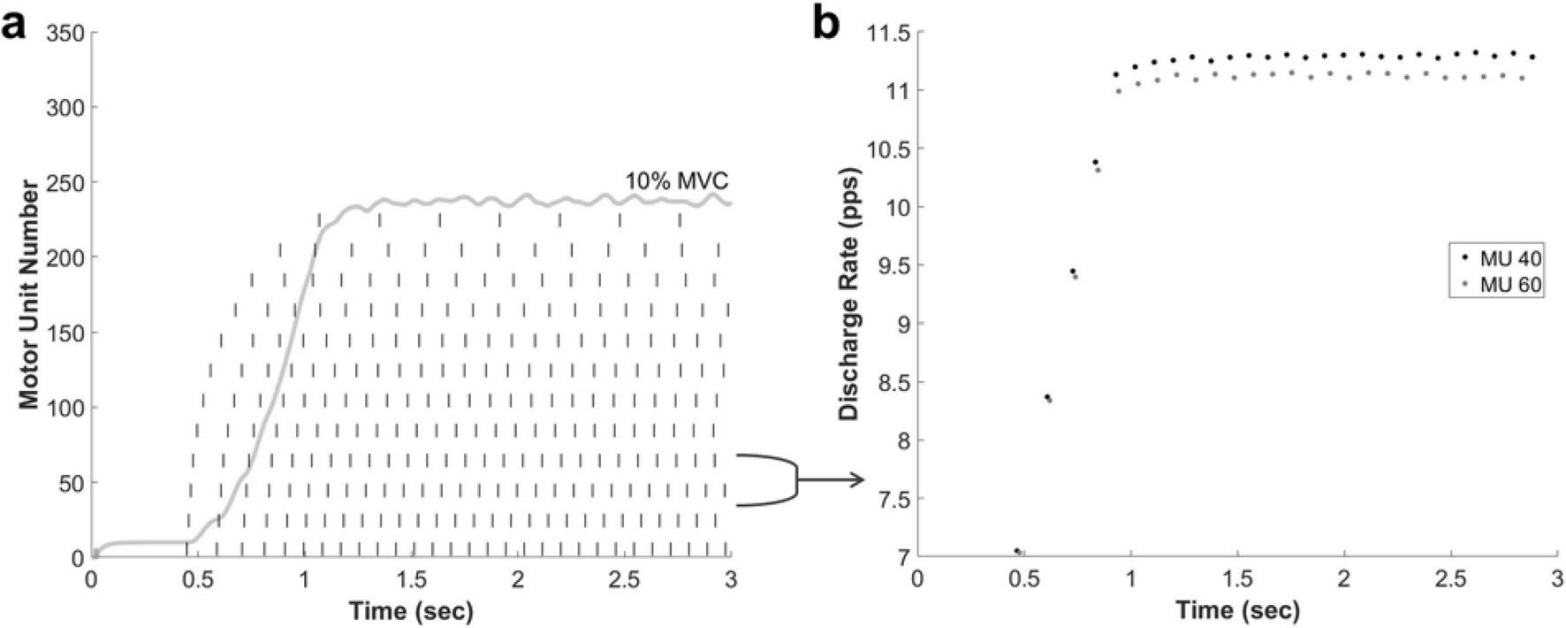
(**a**) Discharge times for every 20th motor unit (dashes) with resulting muscle force output (solid line). The stimulation profile increased linearly for one second until reaching the peak amplitude corresponding to 10% MVC, after which point it was held constant for two seconds. (**b**) Discharge rate, in pulses per second, of motor units 40 and 60 over the course of the simulation, showing the relationship between intensity and discharge rate to demonstrate rate coding.

The neuronal network exhibits rate coding based upon the discharge rates of each motor unit, shown for two representative motor units (Fig. 4). Below the minimum discharge rate (6.78 nA), no spiking occurs. After the minimum discharge rate, there is a linear relationship between stimulation intensity, represented by an increase in amplitude of the applied current, and the discharge rate. This relationship continues until the peak discharge rate is reached, after which point the discharge rate has little variation.

### Incorporation of tissue mechanics predictions

**Figure 5:**
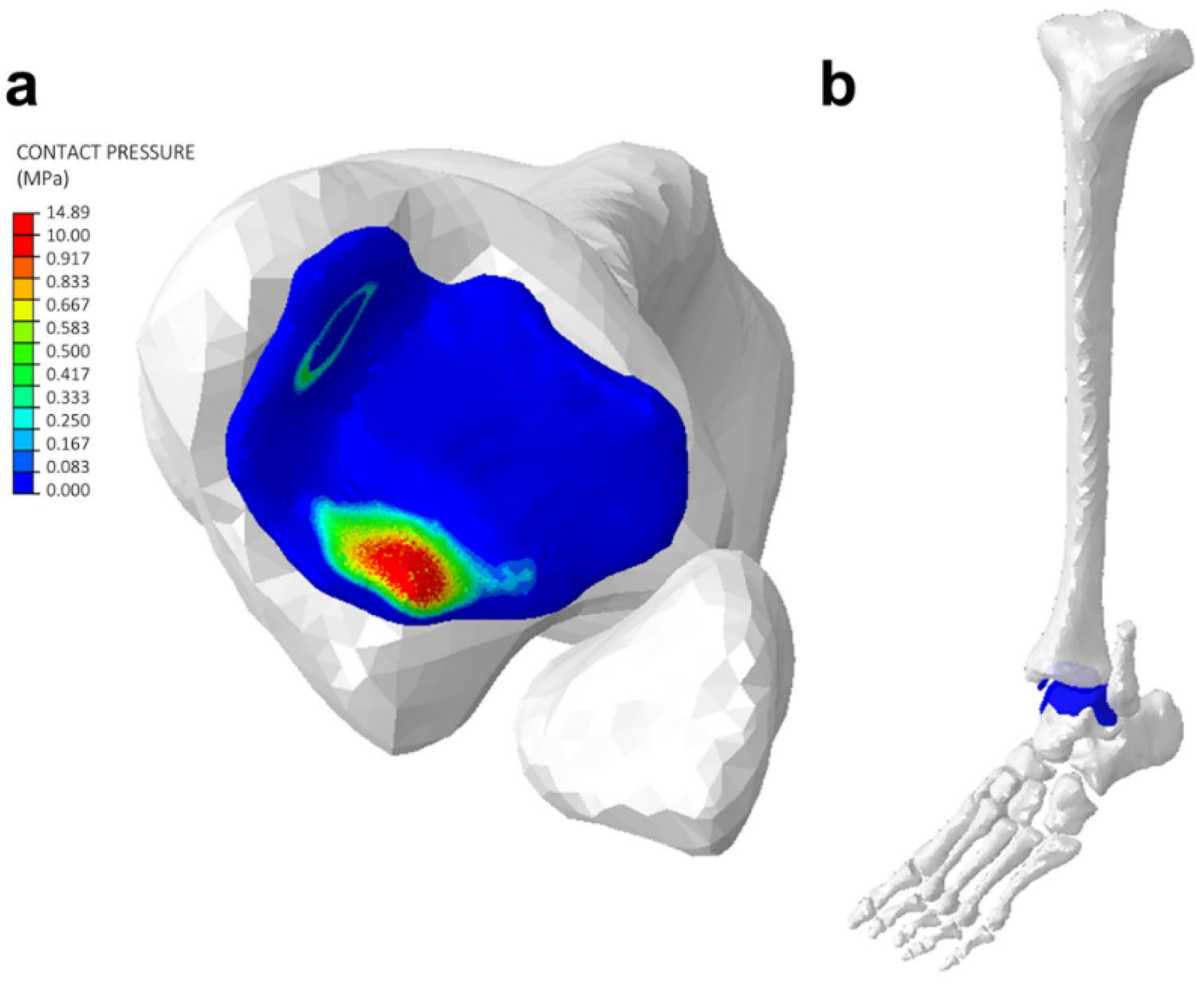
(**a**) Contour map from Abaqus (version 2020; https://www.3ds.com) simulation integrated with NEURON (version 7.7.2) showing contact pressure on the tibia articular cartilage during ankle plantarflexion. The region of higher contact pressure is located posteriorly. (**b**) Plantarflexed position of the tibia-talus joint.

The contact pressure between tibial and talus articular cartilage during ankle plantar flexion was measured throughout the simulation (Fig. 5). The peak pressure achieved during the simulation was 14.89 MPa. The inclusion of cartilage and contact interaction in the integrated model demonstrates the ability of the model to perform more complex biomechanical analyses than is possible using rigid body simulations.

## Discussion

The direct agreement between the muscle force output from Kim^19^ and the single motor neuron FE NMS model verifies that the NEURON model has been accurately integrated with the Abaqus FE environment. The capability of the integrated NMS model with neuronal network to exhibit the principles of motor unit recruitment and rate coding show that the model accurately simulates the neural drive to muscles.

The independent computation times for the NEURON and Abaqus components of the FE NMS model highlight the ability to increase complexity in either component without modifying the run time in the other. A benefit of using NetPyNE to scale the neural architecture to be more representative of physiological muscle is that the software has been designed to run parallelized simulations, which in future models will increase efficiency of large-scale neuronal networks.

The efficacy of this model to accurately simulate various neural commands at different muscle force levels was shown through the verification of the principles of motor unit recruitment and rate coding. This illustrated the ability of the NMS model to accurately simulate skeletal muscle forces needed to drive in vivo movement. It was shown that the NMS model is capable of robust neural architecture scaling, and is therefore applicable to muscles of all sizes throughout the body.

The interspike intervals presented at 10% MVC (Figs. 2d, 3d) are slightly lower than those reported by Thompson et al.^22^ for soleus motor unit spontaneous discharges, but spontaneous discharges would be more variable, and therefore have longer interspike interval times than stimulated motor units. Also, the decrease in interspike interval with an increase in intensity is physiologically accurate across both stimulation profiles (Fig. 2, 3) because discharge rate increases with intensity resulting in a decrease in time between subsequent discharges. In the ramp-up and ramp-down stimulation profile (Fig. 3), there was an asymmetry in discharge rates between recruitment and de-recruitment of motor units, as was shown to be the case in soleus motor units during experimental recordings^23^.

The average motor neuron diameters within recruitment threshold ranges were calculated to verify the motor unit size distribution in the neuronal network. The average diameters were comparable to previously published values^24^, showing that the recruitment threshold distribution occurring due to the exponential diameters of the motor units matched in vivo values. The discharge rates at 10% MVC ranged from 7.03 to 11.28 pulses per second (pps) (Fig. 4). These values are within the range found for motor unit discharge rates at recruitment and peak force^25^.

The model developed here has a similar neural architecture to previously developed fully predictive NMS models^10–17^. The neuron geometry in this model was reconstructed from a cat spinal motor neuron^19^, which is more complex and physiologically accurate than previous models which built two-compartment cell models^13,14^. The most similar model is the five-component model (motor neuron pool, muscle spindles, half-sarcomere, fiber, and continuum mechanics) of Heidlauf and Röhrle^11^. Our model incorporates a program designed specifically for neuronal network simulations, rather than using a general bioengineering software^11^. This has potential benefit because it is easier to create larger, complex neural architectures, as exhibited here with a 310 motor neuron pool compared to 10 in prior literature^11^. This can be accomplished with NetPyNE, as was done in the motor unit recruitment and rate coding verification, since it was designed to facilitate the development of large neuronal networks using NEURON.

In this study we presented a FE model with a simplified representation of the ankle with two point-to-point muscles to serve as proof-of-concept that a NEURON simulation can be integrated with a FE environment to create a fully predictive NMS model. Musculoskeletal model complexity in the isometric contraction simulations used for verification of software integration is similar to that of existing NMS models with rigid-body musculoskeletal representation^15–17^. The inclusion of contact interaction at the tibia-talus joint takes the analysis a step further to demonstrate that additional FE model complexity can easily be incorporated within our integrated FE NMS environment.

Abaqus is frequently used for more complex musculoskeletal simulations, including the use of 3D muscle geometries and sophisticated biomaterial models^26–30^. Future work on this model will focus on incorporating these components so that the FE NMS model may be extended to perform more complex biomechanical analyses that better capture physiological interactions and dependencies between the nervous and musculoskeletal systems. Additionally, the neuronal network developed in this study will facilitate future work with complex 3D muscle architectures because the current network can be minimally modified to include muscle fiber innervation.

The scope of this work was limited to verifying integration between the software platforms and the resulting muscle force generation from the FE NMS model. Limitations of the current model are the simplicity of the musculoskeletal model, lack of validation against kinematic data, and neural signal only including input from motor neurons. The complexity of the FE model should be increased in future work to incorporate 3D representations of musculature and ligaments and validate the resulting human motions against experimental data. Additionally, the NEURON simulation should be expanded to include additional cell types representative of electrical signals generated in the brain necessary to study neurodegenerative disorders.

This is the first time that a predictive neural architecture has been integrated into a musculoskeletal finite element environment. A fully predictive NMS model capable of running within a FE environment, as presented in this work, can aid in improving our understanding of how the neural and musculoskeletal systems work together to generate and control movement in both healthy and pathological individuals. In the future, this model may be applied to study neurodegenerative and neurodevelopmental movement disorders.

## Methods

The design approach for the NMS model was to develop an accurate representation of nerve-muscle interaction that would mimic in vivo muscle activation. To do this, the slow motor unit model developed by Kim^19^ in the NEURON simulation environment (version 7.7.2) was modified to generate a motor neuron pool consisting of 310 motor units and incorporated into a FE musculoskeletal model based upon a previously developed model^31^.

**Figure 6:**
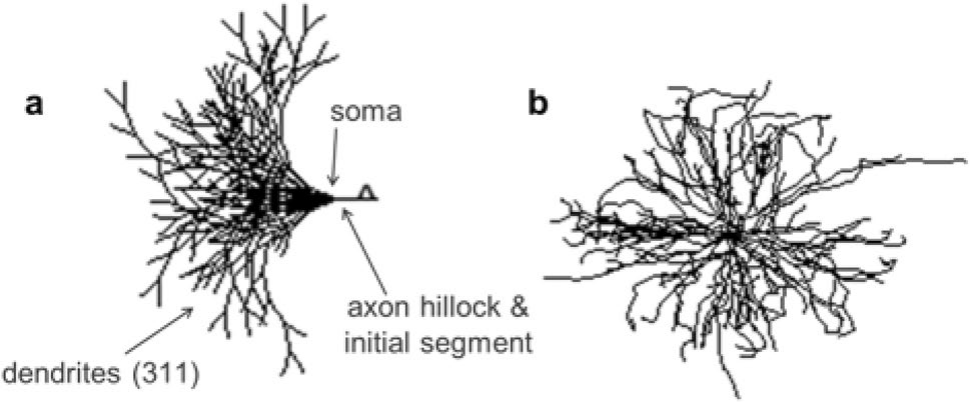
(**a**) 2D and (**b**) 3D representations of alpha motor neuron.

The neuromuscular model developed by Kim consists of a single motor neuron innervating a cat soleus muscle^19^ and is publicly available on ModelDB^32^. The alpha motor neuron has 311 dendrites connected to the soma, which is then connected to the axon hillock and initial segment (Fig. 6). The 3D neuron geometry was reconstructed from scans of a cat spinal motor neuron^19^. All cellular components exhibit passive properties, and the soma, dendrites, axon hillock, and initial segment also include various ion channels for active property definitions. The potassium (delayed rectifier, calcium-activated) and sodium (fast, persistent) channels elicit spiking in all active cells, and the calcium channels (N-type, L-type) play a vital role in bursting activity that elicits force generation in muscles required for movement. The model of the neuromuscular junction includes components for calcium dynamics, activation dynamics, and force production. The force production is based on a Hill-type muscle model with active and passive force generating elements^19^.

**Figure 7:**
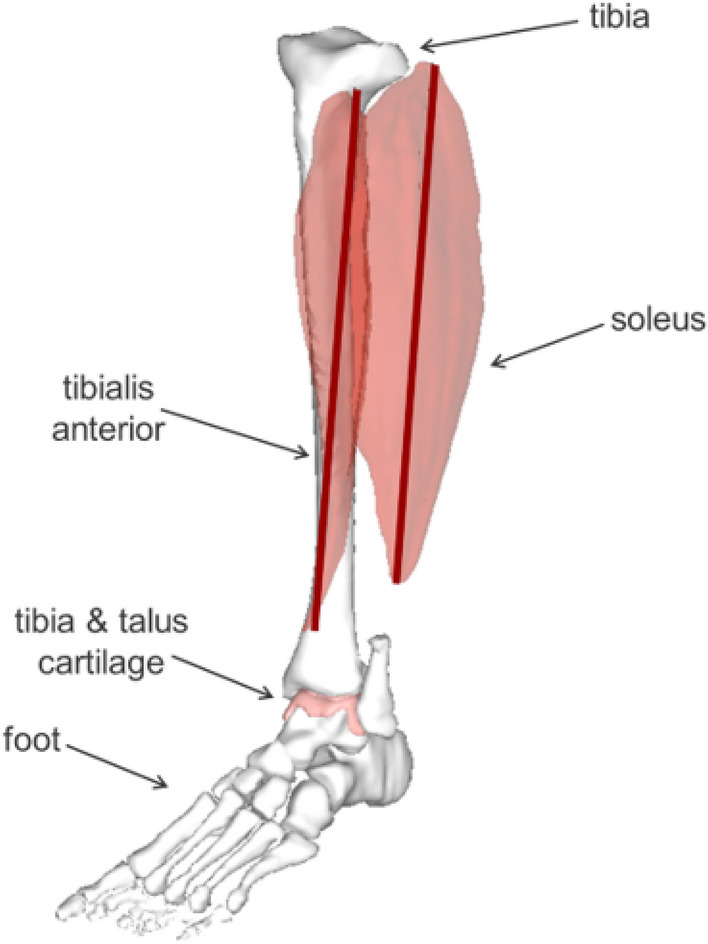
Abaqus musculoskeletal model of the ankle joint including geometries of the bones, muscles, and cartilage.

The musculoskeletal model is a simplified representation of a human ankle joint (Fig. 7). All geometry in the model was segmented from the Visible Human Male dataset^33^. The model includes the soleus and tibialis anterior muscles represented as axial connectors positioned to run through the centroid of the muscle cross-sectional geometry. The model also includes the foot bones, tibia, and 3D articular cartilage^31^ at the tibia-talus joint. Muscle contraction is controlled by applying the forces from the NEURON simulation calculations to the soleus axial connector. Neural parameters determined for felines have been shown to share many of the same features as those seen in humans^34^, therefore many NMS models of humans utilize feline neural parameters^13,14,16^, as was done in this study.

**Figure 8:**
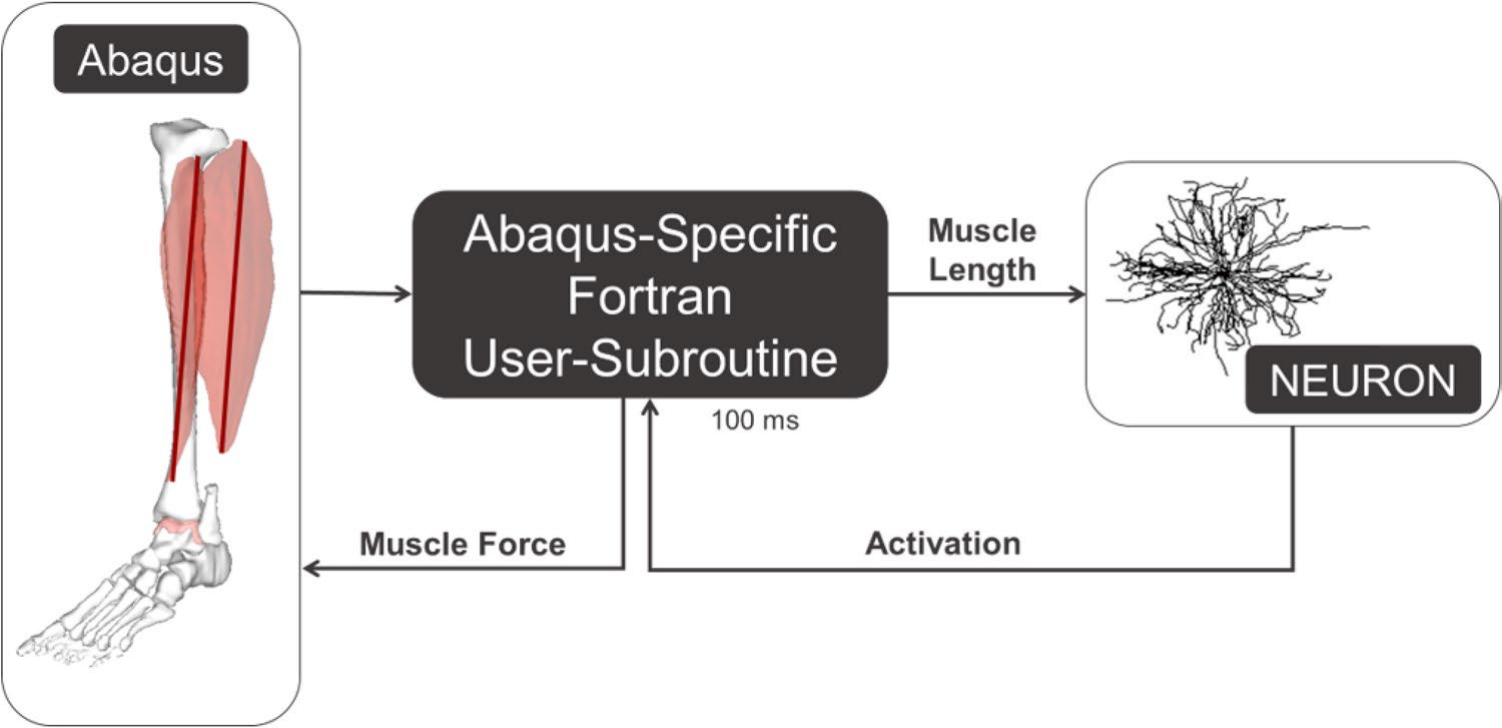
Flow of information in the integrated FE NMS model. A NEURON simulation is performed using a call from the Abaqus-specific Fortran user-subroutine every 100 ms. From that simulation, the activation is input into the muscle force calculation. The force is then applied to the soleus muscle in the Abaqus musculoskeletal model.

All simulations were performed in Abaqus/Explicit, which included a Fortran user-subroutine (vuamp) as an interface between NEURON and Abaqus (Fig. 8). NEURON is called every 100 ms of the simulation by running a Python script from inside the Abaqus-specific Fortran subroutine. During the NEURON simulation, the activation calculated in the calcium dynamics and activation dynamics modules is input into the force calculation. The resulting forces are input back into the Fortran user-subroutine to apply to the soleus muscle connector in Abaqus.

### Verification of software integration

An integrated NMS model containing a single motor neuron in the motor neuron pool was used for verification of the two software environments. The same input stimulation profiles as the Kim motor unit model were used as input into the simulation^19^. The simulated forces from the single motor neuron FE NMS model were then compared to published results (Fig. 1) and the RMSE between the output profiles was calculated.

### Verification of in vivo neural behavior

Motor unit recruitment and rate coding capabilities of the model were demonstrated to show the efficacy of the model to produce muscle forces from neural commands generated from a neuronal network. A neuronal network, or motor neuron pool, was generated using NetPyNE (Networks using Python and NEURON)^35^. NetPyNE was chosen to scale a single neuron into a network of 310 motor units because the program was designed specifically to facilitate the development of large-scale, complex neuronal networks written in NEURON. The diameters of the neurons were varied for motor unit recruitment to occur following an exponential distribution^20^ with a range from 48.8 to 99.7 μm, which is within the diameter range estimated for human motor neurons^16^. The peak twitch force for each motor neuron was calculated using an exponential distribution with a 100-fold range^20^. In the network model, the total muscle force which was applied to the soleus muscle in the FE environment was calculated as the summation of twitch forces from all motor units^20^. A neuronal network of 310 motor units was created to innervate the soleus muscle based on estimates of the total number of motor units per specific muscle in humans and felines^36–39^.

For motor unit recruitment verification, two activation profiles were applied to all motor units uniformly with randomly distributed noise applied independently for each motor unit. Noise was an offset to the stimulation amplitude at each time point in the simulation and was calculated as a random number from a normal distribution with a mean of 0 nA and standard deviation of 0.2 nA. The modeled motor neuron pool was activated to simulate three amplitudes corresponding to 10%, 40%, and 75% of MVC, or approximately 3 N, 12 N, and 23 N, respectively. These values correspond to feline muscle forces, as the original neuromuscular model parameters^19^ were tuned to match those experimental values. The first stimulation profile consisted of a 4 s simulated ramp and hold contraction that increased linearly from baseline amplitude to the target force over a 2 s period and was then held constant for an additional 2 s. The second profile linearly ramped up to the target force and then downward to baseline amplitude, both over a 2 s period. The resulting muscle forces were plotted to ensure they followed accurate muscle behavior^20^. The interspike interval, or the time between each subsequent discharge, for each motor unit was calculated at each force level. Additionally, the recruitment threshold, or force at which each motor unit is recruited, was calculated as a %MVC to verify the motor neuron diameter distribution and orderly recruitment.

To demonstrate rate coding in the integrated FE NMS model, a simulation was performed with a ramp and hold force profile which ramped up to 10% MVC over 1 s, followed by 2 s of constant stimulation intensity. The muscle force level of 10% MVC was chosen for comparison to previously published data^25^. The discharge rate for each motor unit was calculated as the instantaneous frequency^40^ and plotted to ensure an accurate relationship between stimulation intensity and discharge rate.

### Incorporation of tissue mechanics predictions

The integrated NMS model with a network of 310 motor units was used to verify that the integrated model could be used to study human joint biomechanics. The hill-type muscle model parameters were modified to match human levels with 300 N of force applied to the soleus muscle for ankle plantarflexion to occur. The contact pressure between articular cartilage at the tibia-talus joint was measured throughout the simulation.

## Data Availability

The neuromuscular model used here to validate results from the finite element framework was provided by ModelDB (Kim^19^) at the publicly available repository: https://senselab.med.yale.edu/ModelDB/ (Model #235769). The integrated model is also available on ModelDB (Model #267184).
